# Counselling within South Africa’s public health system: Mental healthcare users’ experiences

**DOI:** 10.4102/jphia.v17i1.1776

**Published:** 2026-06-24

**Authors:** Maura Lappeman, Cathy Aaron, Rene Botha, Nasera Cader-Mokoa, Nousheena Firfirey-Brijlal, Fatima Ismail, Vivian Leibrandt, Jonathan Nell, Claire van Dyk

**Affiliations:** 1 Western Cape Department of Health and Wellness, Cape Town, South Africa; 2 Department of Psychiatry and Mental Health, University of Cape Town, Cape Town, South Africa; 3 Department of Psychiatry, Faculty of Health Sciences, Stellenbosch University, Stellenbosch, South Africa; 4 Private Practice, Cape Town, South Africa; 5 School-based Support, Curro Uitzicht, Cape Town, South Africa; 6 Neuroscience Institute, University of Cape Town, Cape Town, South Africa

**Keywords:** registered counsellor, mental health, mental healthcare user, South Africa, primary healthcare, public health, stigma, counselling

## Abstract

**Background:**

South Africa faces a significant mental health treatment gap, with many individuals in need unable to access adequate psychological care. Registered Counsellors (RCs) were introduced to reduce this gap, particularly in impoverished communities, by offering accessible, affordable psychological services.

**Aim:**

This article forms part of a broader study on the integration of RCs into public healthcare and specifically explores the lived experiences of mental healthcare users who received counselling from RCs within the Western Cape Department of Health and Wellness.

**Setting:**

The sampling and interviews were conducted in primary healthcare clinics within the Western Cape Department of Health and Wellness.

**Methods:**

An exploratory qualitative design was employed, using purposive sampling to recruit 10 participants. Semi-structured interviews were conducted and analysed using thematic analysis.

**Results:**

Four major themes were generated: (1) confronting stigma and misconceptions around counselling, (2) uncertainty about the role of RCs, (3) developing agency and self-reflection through counselling, and (4) recognising the transformative impact of counselling. Findings indicate that RCs play a vital role in providing first-line psychological support by creating non-judgemental spaces that promote self-reflection and emotional processing. However, the study also reveals persistent stigma surrounding help-seeking and a limited public understanding of the RC’s role.

**Conclusion:**

These insights underscore the need for clearer role definitions, enhanced public awareness and policy reforms to enhance the integration of RCs within primary healthcare.

**Contribution:**

Enhancing mental health literacy across communities remains essential to achieving equitable and effective service delivery.

## Introduction

Mental healthcare is a fundamental component of overall health, and adequate access remains a global concern. Marked disparities persist between high- and low-income countries, as access to mental health services is largely determined by socioeconomic resources and system capacity.^1,2^ The World Health Organisation (WHO) reported that the mean mental health coverage for individuals diagnosed with serious mental illness was 59.5% in high-income countries compared to only 10.9% in low- and middle-income countries.^3^ This inequity has been consistently documented^4,5^ and became especially visible during the coronavirus disease 2019 (COVID-19) pandemic, which further exposed the fragility of mental health systems worldwide.^2,6,7,8^ Limited access to care, coupled with low levels of mental health literacy, has also been associated with poor health outcomes and reduced help-seeking behaviours in low- and middle-income contexts.^9^

The disparity in access to mental healthcare services remains pronounced in South Africa despite efforts to enhance rights-based inclusion in healthcare spaces after the end of Apartheid. The country ranks among those with the highest levels of income inequality globally, as reflected in its Gini Coefficient,^10,11^ and evidence indicates that these socioeconomic disparities significantly limit individuals’ access to mental healthcare services.

Evidence shows that individuals residing in low-income communities are more likely to experience mental distress and severe mental illnesses compared to individuals in affluent areas, underscoring the critical need for accessible mental health services in low-income communities.^12,13^ Multiple factors contribute to South Africa’s widening mental health treatment gap, including structural and financial barriers, low perceived need for treatment, limited mental health awareness and pervasive stigma, among other systemic challenges.^14^ Despite the urgency of this crisis, equitable access to mental healthcare remains a distant and unfulfilled imperative in South Africa.

### Mental health professionals in South Africa

Mental health systems globally are facing a significant shortage of trained professionals, especially in low- and middle-income countries. In these regions, the burden of mental health issues is disproportionately high compared to the available resources. There are substantial gaps in treatment globally, which restrict access to preventive, promotive and community-based mental healthcare.^15,16,17^

Across Africa, these global challenges are further intensified by chronic underinvestment in mental health systems, limited integration of mental health services into primary healthcare (PHC), and continued reliance on hospital-based, specialist-led models of care. The continent reports among the lowest densities of mental health professionals globally, with pronounced disparities between urban and rural areas, resulting in limited access to basic counselling and psychosocial interventions.^18,19,20^

In South Africa, the mental health crisis is further compounded by inadequate mental healthcare infrastructure and a severe shortage of qualified, competent mental health professionals.^21^ As of 2019, South Africa’s psychiatrist-to-population ratio is 1.52 per 100 000, with approximately 80% of these specialists concentrated in the private sector. In contrast, certain rural provinces have as few as 0.03 psychiatrists per 100 000 people, reflecting stark inequities in distribution.^22^ Access to psychology services is similarly constrained, with an estimated 1.5 psychologists per 100 000 population in 2022.^23^ Consequently, mental health services within primary care remain constrained, prioritising medication management for individuals with severe mental illness. In contrast, limited provision exists for counselling and psychosocial interventions that target less severe mental health concerns.^14^ This shortage of mental health professionals and resources has resulted in a centralised, urban-oriented system that is predominantly reactive rather than preventive, leaving rural and low-resource communities critically underserved.^24^ The community-based component of South Africa’s mental health system remains underprioritised and underfunded, resulting in a poorly resourced and ill-equipped sector that further restricts access to care.^2^

In the Western Cape, where this study was conducted, these national challenges persist despite relatively better-resourced health services than in other provinces. Mental health services remain concentrated in urban centres, while rural and peri-urban communities continue to experience workforce shortages and limited availability of community-based mental healthcare.^25^ These structural constraints continue to limit early intervention and preventive approaches, reinforcing reliance on specialist-driven services.^18^

South Africa faces a grave shortage across all categories of mental health professionals, including psychosocial workers and non-professional counsellors.^26^ The current reactive system does little to mitigate mental healthcare inequities, highlighting the urgent need for strategic interventions to broaden access and address the population’s mental health needs.^2^ Addressing these challenges will require targeted strategies, including the removal of production caps set by regulatory bodies, permitting private higher education institutions to offer health profession degrees, and expanding the number of funded posts for registered counsellors (RCs) within the public sector.

### Addressing disparities of access

Efforts have been made within the public sector to address the mental healthcare gap. In March 2014, the Western Cape Department of Health and Wellness (WCDoHW) introduced ‘Healthcare 2030’.^27^ Healthcare 2030 is a strategic framework aimed at improving the well-being of the population and creating better health services within the Western Cape health system, making specific reference to counselling as a tool to improve mental well-being. The vision is for the Western Cape population to have access to person-centred quality care. Within the Healthcare 2030 approach, RCs have been identified as playing a crucial role in enhancing the preventive and promotive aspects of mental healthcare.^27^ Counselling also plays a crucial role in mitigating health-risk behaviours that contribute to the overall burden of disease and in alleviating complications associated with existing chronic health conditions (e.g. the exacerbation of hypertension when anxiety symptoms are unmanaged). The RCs occupy a pivotal role in mitigating the mental healthcare gap arising from the shortage of specialist professionals, by delivering primary-level psychological services, particularly in underserved communities.^26^

Subsequently, in 2023, the ‘Make Every Contact Count (MECC): Supporting Self-Management Through Healthy Conversations’ strategy was implemented. This initiative was developed by the WCDoHW to further clarify the role of ‘counselling’ within various healthcare services. The initiative led to the introduction of RCs as key players in providing structured psychological support at the PHC level.^28^

While the professional designation of RCs was officially written into legislation in 2003,^29^ the role only made its way into the public health space after 2020, where RCs were formally introduced to function as a pathway between community health services and specialist mental health services.^28^ The RC registration category was designed to establish a mid-level cadre of practitioners equipped to deliver basic primary and preventative psychological interventions, thereby reducing the demand for specialised psychological services, which remain limited.^30,31^ The inception of RCs in public healthcare makes them particularly valuable in low-income settings.^32^

Although qualitative research on Mental Healthcare Users’ (MHCUs) direct experiences is scarce, current evidence shows that patients consistently value the supportive and non-judgemental environments created by RC.^32,33^ The MHCUs typically describe RCs as helpful in managing stress, bereavement, trauma and everyday psychosocial challenges.^32^ In community-based settings, RCs are thought to reduce stigma and improve access to mental healthcare.^30,33^ Nevertheless, the scarcity of rigorous evaluations reveals a persistent gap in understanding the broader clinical and social impact of RCs. However, the available literature highlights multiple challenges associated with integrating RCs into the broader public health system, demonstrating how these obstacles ultimately constrain their reach and limit their capacity to meaningfully impact have a meaningful impact on communities as originally envisioned for this role.^30,33,34^

The provision of mental health counselling by RCs in underserved PHC settings offers substantial benefits; however, both patients and RCs face numerous challenges. Structural barriers, including the limited formal recognition and integration of RCs within public health facilities, significantly constrain their capacity to reach and serve broader populations.^30,33^ Mental healthcare users reportedly also face difficulties when referrals to higher levels of care are needed, as RCs are sometimes not recognised by more specialist-level healthcare professionals.^35^ The lack of recognition of the competence of RCs, combined with resource limitations, hinders many communities from experiencing the potential benefits that RCs can offer. Registered Counsellors have the potential to play an essential, supportive role in communities; however, those who work in these spaces often do so without adequate backing or access to necessary resources. When afforded the opportunity to engage with communities in a collaborative, ecosystemic manner, RCs can have a meaningful impact, delivering essential psychosocial and psychoeducational support, fostering positive outcomes.^31^

### Amplifying the mental healthcare users’ voice

Listening to MHCUs’ voices regarding their experiences with RCs is vital in enhancing mental health service delivery in South Africa.^36^ Their feedback provides insights into the effectiveness, accessibility and cultural relevance of counselling interventions, thereby enabling continuous improvement and responsiveness to client needs.^37^ Giving MHCUs’ voices a platform may enhance their agency in validating distressing lived experiences, and in turn, support the formation of truly collaborative patient-practitioner relationships.

Given the challenges RCs face in role clarity, incorporating MHCUs’ perspectives can help to further clarify the service impact as well as advocate for the RC professionals’ value within the broader healthcare system.^30^

The implementation of RCs within the public health service presents both challenges and benefits; however, the limited formal research exploring MHCUs’ experiences with RCs exacerbates these challenges. This study aims to provide insights into MHCUs’ experiences of engaging with RCs in PHC settings in the Western Cape, South Africa.

## Research methods and design

### Rationale

The WCDoHW introduced Healthcare 2030, a strategic framework promoting person-centred care, which identified counselling as essential for enhancing well-being.^27^ The 2023 MECC strategy operationalised this by integrating RCs to provide structured psychosocial support at the PHC level.^28^ While RC inclusion in public health aims to relieve the pressure on an already strained healthcare system, there is currently limited research on the impact of these interventions from the perspective of service users. This study addresses this gap by exploring how MHCUs experience RC services within PHCs in the Cape Metropole, a gap which, if not explored, may result in an ineffective service being offered.

### Study aims

The primary aim of this study is to explore the integration and perceived impact of registered counselling services within the Cape Metropole from the perspective of MHCUs. The study seeks to determine if the inclusion of RCs has effectively translated into accessible, high-quality mental health support for users at the PHC level.

### Study objectives

To achieve the primary aim, the following objectives were established:

- To explore the current state of the registered counselling services available to MHCUs within the Cape Metropole.
- To examine the extent to which MHCUs perceive counselling as integrated into their overall healthcare framework.
- To explore the impact of registered counselling interventions on MHCU outcomes – specifically mental health and general well-being.
- To identify barriers encountered by MHCUs when accessing or utilising registered counselling services.
- To provide user-centred recommendations for enhancing the utilisation of RCs within the WCDoHW to improve mental health service delivery.

### Methods and setting

This study used an exploratory qualitative design to explore the experiences of MHCUs engaging with counselling services within the WCDoHW. This design was specifically chosen for its flexibility in exploring this relatively new service. It allowed the researchers to probe beyond individual experiences to identify systemic facilitators and barriers affecting MHCUs in a high-pressure environment. By utilising this approach, the study could capture the multi-dimensional nature of service delivery, providing the depth required to generate evidence-based recommendations for policy refinement and improved mental health service delivery. This approach also captured the nuanced, subjective perspectives of MHCUs, allowing for an in-depth understanding of their perceptions, meanings and lived experiences in accessing mental healthcare.^31^ In the South African PHC context, where services are often under-resourced and patient experiences are influenced by socioeconomic, cultural and systemic factors, qualitative methods are valuable to uncover the barriers, facilitators and personal significance of counselling interventions. By focusing on personal narratives, the design facilitated rich, detailed insights into the perceived benefits and challenges of RC-led counselling services in public health facilities.

### Participants and sampling

Participants were selected via purposive sampling to ensure the inclusion of individuals who saw the RC for more than one session, as their experiences were central to addressing the research questions.^32^ To ensure ethical recruitment and minimise perceived coercion, initial contact was made with the participants by the RCs or mental health nurses at each site. These clinicians identified eligible participants and briefed them on the study’s objectives. If an MHCU expressed interest in participating, their contact details were shared with the research team with the MHCU’s explicit permission. The researchers then contacted potential participants to conduct a full informed consent process in the participant’s home language. It was made clear to all potential participants that they would not be disadvantaged in any way should they decline to participate.

The 10 participants were recruited from five Community Health Clinics and three District Hospitals across Cape Town, where RCs are based (see Table 1). The sample was gender-skewed, with most participants being female. All participants were over 18 years old and provided informed consent in their home languages. For the study, MHCUs were required to speak English, Afrikaans and isiXhosa (the three official languages of the Western Cape).

**TABLE 1:** Mental healthcare users who received counselling from registered counsellors in primary healthcare (*N* = 10).

Participant	Age (years)	Sex	Reason for referral	Number of 30 min – 60 min sessions
1	55	Female	Marital stressors	Monthly for 2 years
2	41	Female	Depressive symptoms	7 Sessions (2022)
			Anxiety symptoms	8 Sessions (2024)
3	51	Female	Adjustment	4 Sessions
4	47	Female	Grief counselling & depressive symptoms	8 Sessions
5	58	Female	Post-trauma symptoms	5 Sessions
6	20	Female	Self-harming behaviours & depressive symptoms	4 Sessions; Admission; Monthly sessions thereafter
7	30	Female	Depressive symptoms & medication compliance	6 Sessions
8	28	Female	Depressive symptoms	5 Sessions
9	49	Male	Post-trauma symptoms	4 Sessions
10	52	Female	Family dynamics	4 Sessions

### Data collection

All semi-structured interviews were conducted in English and Afrikaans, as preferred by participants. The researchers made provision for isiXhosa-speaking MHCUs; however, no participants required a translator to communicate during the data collection process. The interviews took place on video call, or in a private consultation room at the clinics or District Hospitals to accommodate participant availability and logistical constraints. Virtual interviews were conducted on the WCDoH clinics or the hospital’s computers in a private consultation space using Microsoft Teams. To maintain digital security, all Microsoft Teams meetings were end-to-end encrypted, and recordings were immediately transferred to a password-protected drive.

All interviews were recorded and manually transcribed verbatim. Maintaining the integrity of analysis and the value of language in multilingual or translated data is essential for ensuring the accuracy and reliability of research findings. Most of our researchers are proficient in both English and Afrikaans, enabling them to proceed with coding as outlined below. The interview guide was structured to align directly with the study’s core research objectives. The initial interview questions were piloted, leading to refinements of the initial question set. Additionally, a series of semi-structured probes was integrated to facilitate a deeper investigation into how participants navigate the implementation of RC services within the WCDoHW

Data were stored on password-protected devices. Participants’ identities were protected through pseudonyms, and only the research team had access to the data. Transcripts were reviewed by all authors, though not all authors participated in every interview.

### Data analysis

After collecting data from 10 participants, no new themes or insights were presented, indicating that data saturation had been reached. Thematic analysis was used as a structured yet flexible framework for exploring participants’ subjective experiences.^38,39^ This process involved a structured six-phase process to ensure a rigorous and systematic interpretation of the data:

- **Familiarisation:** Transcripts were read and re-read by the primary researchers while listening to the original audio recordings to ensure semantic accuracy and immersion in the participants’ narratives.
- **Initial coding:** Systematic, manual coding was conducted across the entire dataset. Codes were generated to capture both semantic meanings and latent ideas regarding the RC-client relationship.
- **Generating initial themes**: Codes were collated and organised into broader candidate themes that reflected recurring patterns across the five community health centre (CHC) and three District Hospitals.
- **Reviewing themes:** To ensure inter-rater reliability, an independent researcher coded the data. The research team then met to compare these independent codes against the original extracts to ensure the themes accurately represented the dataset.
- **Defining and naming themes:** Minor discrepancies were resolved through collaborative discussion until a consensus was reached. Themes were refined to ensure they were distinct and addressed the research questions directly.
- **Reporting:** The final thematic structure was integrated into a coherent narrative, supported by verbatim quotes in English and Afrikaans (with English translations) to maintain the participants’ ‘authentic voices’.

To ensure trustworthiness, the researchers each maintained a reflexive journal throughout the study, supporting the co-construction of interpretations about the effectiveness and role of RCs within the healthcare system.^40^ Participant responses were triangulated through a systematic review of interview notes, enhancing credibility and promoting reflective engagement with the data.^38^

### Ethical considerations

Ethical clearance to conduct this study was obtained from the University of Cape Town Faculty of Health Sciences Human Research Ethics Committee (No. 575/2023) and the Western Cape Department of Health and Wellness (No. WC_202211_034).

#### Ethical principles observed

The ethical principle of confidentiality was maintained^41^ through the use of pseudonyms (e.g. Participant 1, Participant 2), and only the research team had access to the raw data – all of whom are Health Professions Council of South Africa (HPCSA)-registered practitioners. The ethical principles of non-maleficence and beneficence were upheld in that the participants were informed of both the risks and benefits of participating through the informed consent process. The informed consent process included participants reviewing an information sheet that outlines the study’s purpose, procedures, risks, and benefits, after which the participants had the opportunity to ask the researchers any questions before deciding whether to participate. Participants were also offered the use of a translator, however, no one requested this. Participants were given the opportunity to retract their interview from the study at any point in time, and participation was voluntary, with no negative consequences for declining.

#### Protection of personal information act and participant privacy

All electronic transcripts were stored on password-protected computers in compliance with Section 19 of *Protection of Personal Information Act (POPIA)* in South Africa.^42^ Identifying information was removed, and data were retained in accordance with institutional and legal requirements. Interviews were conducted in private consultation rooms or online via video call, and all data was stored on password-protected devices. Any discussions regarding the study were done on end-to-end encrypted applications or via the Western Cape Government email platform, which is also password-protected.

#### Data storage and protection

The original data has not been shared with publishers because of its confidential nature, and it has been archived on a password-protected device, in accordance with POPIA Section 14. The data will not be used for further research, in accordance with Section 15 of POPIA – unless additional consent is obtained from the participants.^42^

### Trustworthiness and rigour

Dependability was maintained through a comprehensive audit trail and a detailed process journal. To ensure credibility, researchers employed active listening techniques, such as paraphrasing and reflecting, during the data collection process. Furthermore, findings were triangulated by cross-referencing interview notes and transcripts, ensuring that the analysis was grounded in converging participant accounts rather than isolated narratives.

Transferability was supported by providing detailed descriptions of the participants, setting and counselling process, enabling readers to determine the applicability of findings to similar public health contexts.

Confirmability was addressed by maintaining a clear distinction between the researchers’ professional experiences and the participants’ perspectives. This was facilitated through regular peer debriefing sessions where the research team reflected on their own biases. An independent researcher audited the entire process to further validate the study’s confirmability.

Authenticity was ensured by presenting participants’ voices through direct interview quotes with minimal editing, reflecting their perspectives rather than researcher’s interpretations.

#### Researcher positionality and reflexivity

The research team comprised HPCSA-registered healthcare professionals from diverse demographic and clinical backgrounds, including RCs, an occupational therapist, and clinical psychologists. This ‘insider’ status provided a nuanced lens through which to interpret the systemic challenges of RC implementation, such as resource limitations and burnout.

However, the team reflexively acknowledged potential biases, particularly a tension between advocating for the RC role and concerns regarding the blurring of professional scopes. Researchers engaged in ongoing bracketing to mitigate the influence of their professional authority and demographic privileges on the interpretation of MHCU experiences. This was done through the use of individual reflective journalling and attending regular group discussions with the research team.

## Results

Through the thematic analysis of the 10 participant narratives (see Table 1), four key themes were identified that illustrate the complex journey from initial resistance to psychological empowerment (see Table 2). These findings suggest that the path to accessing mental health support in the PHC is not a linear one, but rather a transition shaped by deep-seated community stigmas and poor mental health literacy. Initially, many participants viewed seeing an RC as something shameful, which was further complicated by a pervasive uncertainty regarding the specific roles of RCs compared to other clinicians. However, as counselling with the RC continued, a shift occurred where their uncertainty was replaced with increased personal agency and self-reflection. Ultimately, these themes reveal a transformative impact, where counselling is reframed from a sign of weakness into a courageous, life-changing tool for navigating daily human struggles in South Africa.

**TABLE 2:** Overview of generated themes.

Theme	Description
1	Confronting stigma and misconceptions around counselling
2	Uncertainty about the role of registered counsellors
3	Developing agency and self-reflection through counselling
4	Recognising the transformative impact of counselling

### Theme 1: Confronting stigma and misconceptions around counselling

Mental Healthcare Users described counselling as a complex and often stigmatised process. Several participants noted that seeking counselling was only for those who were ‘mad’ or had ‘lost it’. In PHC settings, such misconceptions foster avoidance and resistance to accessing counselling, which reflects broader community misunderstandings about the purpose of counselling. These sentiments are illustrated in the following quote. ‘The minute you say you go for counselling, people think that you’ve lost it. You’ve lost your mind, and you can’t cope. But we all can’t cope’ (Participant 9, male, 49-years old). Stigma was further described as a barrier to seeking mental healthcare, as MCHUs often avoided the counselling process. When counselling is viewed as a sign of ‘losing your mind’ rather than a form of help-seeking to improve mental distress, MHCUs resisted getting help even when struggling. The reflection that ‘we all can’t cope’ challenges this stigma by framing mental distress as universal and not as personal failures. However, the recognition of mental distress as universal does not translate into having the motivation to seek help, given the negative gaze linked to the counselling process. To avoid the stigma, MHCUs explored alternative avenues for seeking mental healthcare. Participants reflected that they felt more comfortable consulting community-based or faith-based services to talk about their distress. Participants reported that seeing an RC was either unheard of within their communities or there were negative associations with consulting an RC, further reflecting resistance of the counselling process. This may highlight the structural invisibility of RC services within community mental health systems, reinforcing their marginal position despite policy integration. Most MHCUs learned about the availability of RC services only from Mental Health Nurses in PHC clinics and hospitals. Participants noted that they would consult with doctors and nurses before engaging with RCs for counselling services. This is illustrated in the following quote. ‘Before engaging with the RC, I only previously spoke to the doctor. I didn’t know anything about RCs before being referred to one’ (Participant 1, female, 55-years old).

The sample demonstrated limited mental health literacy and minimal prior exposure to RCs, both of which contributed to participants’ initial resistance to seek help. The unfamiliarity and limited mental health literacy regarding RC services and where to seek help in PHC also generated further uncertainty about what to expect during consultations with RCs and how to distinguish among various types of mental health professionals.

### Theme 2: Uncertainty about the role of a registered counsellor

Mental Healthcare Users’ narratives blended the roles of mental health professionals without comprehending the key differences between psychiatrists, psychologists and RCs. At times, MHCUs often refer to RCs as doctors. Those who attempted to find key differences further found it challenging to differentiate between mental health professionals, often erroneously associating certain roles and functions. Furthermore, participants understood that a Psychologist, an RC and a Psychiatrist are professionals who offer counselling services to the general population. Additional misconceptions about the role and function of different mental health professionals often led to misunderstandings about who can prescribe medication and who provides counselling and psychotherapeutic services. The pervasive sense of role confusion is demonstrated in the following quote. ‘I wouldn’t even know the difference between an RC and a psychologist’ (Participant 3, female, 51-years old). ‘One [*psychologist*] prescribes you medication and the other [*RCs*] is the person you speak to and load off’ (Participant 7, female, 30-years old).

Following the confusion of the different roles was the uncertainty about what to expect, which pervaded MHCUs experiences and often elicited further confusion about RC services. All MHCUs reported that upon entering the counselling space, they did not have a clear sense of how an RC can help with their mental distress. This was further illustrated by the following quote:

‘[*T*]he sister [*Mental Health Nurse*] said she would refer me to the RC, [*and*] I just went because she said I must go. I didn’t expect much or think it would help.’ (Participant 8, female, 28-years old)

Interestingly, participants’ uncertainty was mostly met with a co-existing sense of hope and an experience that they appreciated as helpful. Mental Healthcare Users’ initial resistance and avoidance were replaced with a sense of relief, comfort and safety after seeing the RC. They further experienced improved clarity as to the roles and functions of various mental health professionals after receiving counselling.

Participants described their RC as straightforward and direct in offering support, while also maintaining a sense of support, presence and gentleness. This often led to drawing comparisons between the different healthcare providers. The following quote exemplified this sentiment. ‘I didn’t feel like the doctors focused on me like the counsellors. The counsellors are more involved in understanding what is really bothering you’ (Participant 5, female, 58-years old).

### Theme 3: Developing agency and self-reflection through counselling

Upon learning the role and functions of RCs in PHC settings, MHCUs grew increasingly used to the counselling process and developed a deeper sense of appreciation for RC services. Participants perceived RCs as non-judgemental and valued the opportunity to speak without self-censorship. Counselling fostered a sense of comfort and hope in alleviating psychological distress. Although some participants initially anticipated a directive, advice-oriented approach, they reported experiencing increased agency and shared responsibility in addressing their mental health, complemented by psychoeducational guidance from the RCs, where appropriate. The counselling space was often characterised as a quiet, introspective environment that allowed for self-reflection and emotional processing. This was noted in all participants and is illustrated in the following quote:

‘[*S*]o in in the beginning I was under the impression that I would be receiving advice in a way forward and an instruction book almost on how to deal with what I was going through. But I’ve now learned that it’s just a space where I can come and speak to her and even hear myself say the things that I think all the time. You know the millions of thoughts in my head and just speaking about it, yeah. I’ve come to learn that that was the whole purpose … And not what I initially thought it was going to be.’ (Participant 1, female, 55-years old)

Seeing RCs also corrected personal maladaptive views of the counselling process. Stigma and the misconceptions echoed in the broader communities’ understanding of counselling services shifted as MHCUs experienced the counselling services offered by RCs. Registered Counsellors often allowed unexpected topics to be explored in a safe and non-judgemental space, which was widely appreciated across the sample. Experiences like this are foundational in changing stigmas that persist in these spaces. Participants also reiterated the foundational shifts that may take place in the therapeutic space, especially regarding personal issues related to identity and help-seeking behaviours. These sentiments are represented by the following quote. ‘You come [*to counselling*] for a certain thing. This [*sessions*] was the hijack story, but everything comes out’ (Participant 5, female, 58-years old).

### Theme 4: Recognising the transformative impact of counselling

When probing participants about the impact RCs have on the greater healthcare system, participants provided predominantly positive feedback. Participants felt that RCs are needed more than psychologists, given their proximity to access mental healthcare at the PHC level. Participants also reported that engaging in counselling dramatically changed the trajectory of their lives and assisted them in ways that are life-changing. Two quotes echoed the broader impact of counselling services on the general sample:

‘[*I*]t’s been absolutely life changing for me because … I first thought that she would ask me questions … and then she tells me what to do. But I’ve now learned that what she does is without me even knowing it.’ (Participant 1, female, 55-years old)

‘[*Y*]ou know, so yes, this has been absolutely life changing. Counselling is essentially about what my experience and understanding is. It is basically helping to give me a fresh perspective with an understanding of psychological processes to help provide tools.’ (Participant 4, female, 47-years old)

Change was also not only intrapsychic but observed by others in the immediate environment of the participants. This change was met with the awareness that participants needed to be courageous to engage in counselling, as can be seen in the following excerpt:

‘[*A*] lot of people say to me, oh, you’ve changed, or you know, you’re such a strong person. And then I have to say to them because I’ve had to step out and be courageous and get help. I couldn’t do all of this on my own. I needed help.’ (Participant 10, female, 52-years old)

The findings revealed that participants’ experiences of counselling with RCs were shaped by a complex interplay of stigma, uncertainty, empowerment and transformation. Many initially approached counselling with hesitation because of community stigma and limited mental health literacy, but came to view it as a safe, reflective space that fostered self-awareness and emotional relief. Despite confusion about professional roles, participants ultimately recognised counselling as profoundly beneficial and life-enhancing, particularly within resource-limited PHC contexts.

## Discussion

This study explored MHCUs’ experiences of counselling services delivered by RCs within public PHC settings in the Western Cape. By highlighting the accounts of service users, the findings demonstrate how counselling is understood, utilised and made significant within South Africa’s public health system, while also exposing the systemic conditions that influence these experiences. Participants’ narratives point to counselling as a relational and preventative form of care that holds relevance in PHC contexts characterised by high psychosocial burden and limited access to specialised mental health services.^32,43^

Stigma surrounding mental healthcare remains a well-documented barrier to help-seeking in South Africa and similar low- and middle-income contexts, particularly where psychological distress is associated with weakness, pathology or social failure.^9,44^ Within this study, participants’ accounts reflected ambivalence toward counselling, with services often associated with being ‘mad’, weak or unable to cope. Fear of negative labelling has been shown to deter engagement with psychological support even when distress is significant,^42^ and participants’ narratives in this study demonstrate how such stigma is compounded by limited mental health literacy.

Counselling was frequently misunderstood or approached only once distress had escalated, reinforcing patterns of silent suffering and poorer health outcomes despite the availability of evidence-based psychological support.^45,46^ These findings suggest that, from the perspective of MHCUs, stigma is not only an attitudinal barrier but also a practical one that delays access to care within PHC settings.

Participants’ experiences further revealed uncertainty regarding the role of RCs within the public health system. Many struggled to distinguish between RCs, psychologists and psychiatrists, reflecting limited awareness of professional scopes of practice.^33^ This confusion mirrors broader patterns observed across other healthcare professional categories, indicating a system-level issue rather than an individual or profession-specific shortcoming. From a service user perspective, unclear role differentiation and unstructured referral processes contributed to uncertainty about what counselling involved and when it was appropriate to access such support. Clearer referral pathways and role communication are therefore essential to ensuring that counselling services are both accessible and meaningful at the PHC level.^35^ Although the HPCSA introduced the RC role to expand access to psychological care at the grassroots level and bridge PHC and specialised services,^47,40^ participants’ accounts suggest that insufficient structural integration continues to shape how RC services are experienced and understood.

Despite these barriers, participants described counselling as a process that enabled emotional processing, self-reflection and a sense of agency within a safe, non-judgemental space. These experiences reflect person-centred frameworks underpinning RC training,^39^ and stand in contrast to the brief, symptom-focused consultations that often characterise public PHC encounters. Rather than receiving directive advice, participants experienced counselling as a collaborative process that supported emotional regulation and self-understanding, particularly among those with limited prior exposure to psychological services. From the perspective of MHCUs, counselling thus functioned as an early and supportive intervention addressing psychosocial needs frequently unmet within the biomedical model approach to healthcare.

The impact of counselling services extended beyond intrapsychic change. Participants reported improvements in their emotional resilience, interpersonal functioning and relational dynamics, with some participants noting that even their family or community members observed the positive changes. These accounts are evidence of the broader psychosocial ripple effect that shapes how MHCUs engage with their social environments. Such experiences align with evidence indicating that RCs can deliver effective and culturally responsive interventions at the PHC level, with outcomes comparable to those achieved by psychologists when equivalent services are provided.^32,33^ In South Africa’s public health system, one marked by shortages of specialised professionals and an escalating mental health burden, the participants’ experiences point to counselling as a form of support that is both accessible and responsive within resource-limited settings.^30,43^ With many African countries offering mental health services within similarly constrained healthcare settings, this research may prove valuable on a broader scale, in countries where specialist psychiatric and psychological professionals are also sparsely available.

The participants’ accounts critically highlight the systemic barriers that shaped their experiences of counselling. Poor visibility of RC services, limited availability of services, and RC role uncertainty within public-sector hierarchies were some of the themes generated in this study. These barriers affected continuity of care and influenced the degree to which counselling was accessed and valued. Addressing such barriers requires structural reforms that integrate counselling more clearly into PHC services. Strengthened referral processes, defined supervision pathways, and consistent recognition of RC roles within public health frameworks are just a few of the cornerstones that will determine the success of registered counselling services in PHC settings. From the perspective of the MHCU, such integration is central to ensuring that counselling is experienced as a legitimate and sustained component of public mental healthcare rather than a peripheral or temporary offering.

## Conclusion and recommendations

The narratives presented in this article illuminate both the transformative potential and the systemic constraints associated with the integration of RCs within the WCDoHW. Participants’ accounts reflected the profound psychosocial impact of counselling, through enhancing emotional regulation, self-understanding, and resilience, while simultaneously revealing the barriers that persist within the broader public healthcare context. The findings suggest that when counselling services are made available and accessible, they hold significant promise for improving emotional well-being, strengthening help-seeking behaviours, as well as reducing the pervasive stigma surrounding mental healthcare in community settings.

Despite these encouraging outcomes, the study also underscores the structural inequities that continue to limit the reach and visibility of RCs. The confusion surrounding professional roles, limited mental health literacy among MHCUs, and uneven distribution of human resources all contribute to a fragmented system that struggles to meet the psychological needs of South Africans. Addressing these gaps requires not only the strategic inclusion of RCs at the PHC level but also sustained investment in public education, supervision and interprofessional collaboration.

### Strengths and limitations

In reflection, there were several strengths to this article. Having MHCU participants recall their first-hand experiences of counselling in PHC settings was invaluable, as this ensured that the data was grounded in truly authentic lived experiences. This approach provided researchers with nuanced insight into the way that RC services are experienced in practice, rather than merely relying on data based on statistics or clinical observations. The qualitative nature of the article was intentional on the part of the researchers, as they wanted to capture the depth of MHCU participants’ real-world experiences, which would have been lost had a more quantitative approach been adopted. It was further advantageous that the researchers themselves are all HPCSA-registered clinicians working in the mental health space, giving them an added level of clinical comprehension through which to analyse the responses of the participants.

However, several limitations should also be recognised. The small, purposively selected sample limits the generalisability of the findings beyond the context of the Cape Metropole. Additionally, participants were recruited from facilities where RC services were already established, which excludes the perspectives of those who do not yet have access to PHC counselling services. Finally, as participation was voluntary, it is important to consider that the sample may unintentionally reflect more favourable accounts of counselling experiences. The findings, therefore, represent subjective experiences within a specific context and should be interpreted accordingly.

### Recommendations

Future research should build upon the findings of this article by exploring the long-term impact of the RC-led interventions, with attention to their impact on clinical improvement, stigma reduction and service utilisation over time. There is currently limited longitudinal research on the outcomes of RC-led interventions. Furthermore, incorporating perspectives from multidisciplinary teams (including nurses, psychologists, and social workers) will be vital in developing an integrated framework for sustainable mental health service delivery. Strengthening the structural position of RCs in public healthcare may thus represent a pivotal step toward realising more equitable, accessible and person-centred mental healthcare in South Africa.

In summary, this article reaffirms the value of person-centred, community-based counselling as a means of humanising care and fostering psychological agency. In contexts where psychiatric and psychological services remain scarce, RCs act as a bridge – one that connects individuals to early intervention and ongoing psychosocial support. Their work alleviates emotional distress at a primary level and continues to enhance the overall responsiveness of the mental healthcare system through appropriate referrals.

## References

[1] Lora A, Hanna F, Chisholm D. Mental health service availability and delivery at the global level: An analysis by countries’ income level from WHO’s Mental Health Atlas 2014. Epidemiol Psychiatr Sci. 2020;29:e2. 10.1017/S2045796018000830 28287062

[2] Shishana O, Stein DJ, Zungu NP, Wolvaardt G. The rationale for South Africa to prioritise mental health care as a critical aspect of overall healthcare. Compr Psychiatry. 2024;130:152458. 10.1016/j.comppsych.2024.152458 38320345

[3] Jaeschke K, Hanna F, Ali S, Chowdhary N, Dua T, Charlson F. Global estimates of service coverage for severe mental disorders: Findings from the WHO Mental Health Atlas 2017. Glob Ment Health. 2021;8:e27. 10.1017/gmentalhealth.2021.24 PMC8320004 34367650

[4] Moitra M, Owens S, Hailemariam M, et al. Global mental health: Where we are and where we are going. Curr Psychiatry Rep. 2023;25(7):301–311. 10.1007/s11920-023-01413-y 37256471 PMC10230139

[5] Giusto A, Jack HE, Magidson JF, et al. Global is local: Leveraging global mental-health methods to promote equity and address disparities in the United States. Clin Psychol Sci. 2024;12(2):270–289. 10.1177/21677026221125715 38529071 PMC10962902

[6] Saltzman LY, Lesen AE, Henry V, Hansel TC, Bordnick PS. COVID-19 mental health disparities. Health Secur. 2021;19(S1):S5–S13. 10.1089/hs.2020.0144 34014118

[7] Maffly-Kipp J, Eisenbeck N, Carreno DF, Hicks J. Mental health inequalities increase as a function of COVID-19 pandemic severity levels. Soc Sci Med. 2021;285:114275. 10.1016/j.socscimed.2021.114275 34365069 PMC8417401

[8] Van Heerden AC, Pozuelo JR, Kohrt BA. Global mental health services and the impact of artificial intelligence-powered large language models. JAMA Psychiatry. 2023;80(7):662–664. 10.1001/jamapsychiatry.2023.1343 37195694

[9] Renwick L, Pedley R, Johnson I, et al. Mental health literacy in children and adolescents in low- and middle-income countries: A mixed studies systematic review and narrative synthesis. Eur Child Adolesc Psychiatry. 2024;33(4):961–985. 10.1007/s00787-022-01997-6 35570227 PMC11032284

[10] World Bank. Inequality in Southern Africa: an assessment of the Southern African Customs Union [Internet]. Washington (DC): World Bank; 2022 [cited 2025 Oct 25]. Available from: https://documents1.worldbank.org/curated/en/099125303072236903/pdf/P1649270c02a1f06b0a3ae02e57eadd7a82.pdf

[11] Mdingi K, Ho SY. Income inequality and economic growth: An empirical investigation in South Africa. Cogent Econ Financ. 2023;11(2):2230027. 10.1080/23322039.2023.2230027

[12] Achoki T, Sartorius B, Watkins D, et al. Health trends, inequalities and opportunities in South Africa’s provinces, 1990–2019: Findings from the Global Burden of Disease 2019 Study. J Epidemiol Community Health. 2022;76(7):585–597. 10.1136/jech-2021-217480 PMC8995905 35046100

[13] Burns JK, Tomita A, Lund C. Income inequality widens the existing income-related disparity in depression risk in post-apartheid South Africa: Evidence from a nationally representative panel study. Health Place. 2017;45:10–16. 10.1016/j.healthplace.2017.02.005 28237744 PMC5438466

[14] Jacobs Y, Myers B, Van der Westhuizen C, Brooke-Sumner C, Sorsdahl K. Task sharing or task dumping: Counsellors experiences of delivering a psychosocial intervention for mental health problems in South Africa. Community Ment Health J. 2021;57(6):1082–1093. 10.1007/s10597-020-00734-0 33161458 PMC8217044

[15] Vigo D, Thornicroft G, Atun R. Estimating the true global burden of mental illness. Lancet Psychiatry. 2016;3(2):171–178. 10.1016/S2215-0366(15)00505-2 26851330

[16] Patel V, Saxena S, Lund C, et al. The Lancet Commission on global mental health and sustainable development. Lancet. 2018;392(10157):1553–1598. 10.1016/S0140-6736(18)31612-X 30314863

[17] Charlson F, Van Ommeren M, Flaxman A, Cornett J, Whiteford H, Saxena S. New WHO estimates on depression and other common mental disorders. Lancet. 2017;390(10100):858–859.

[18] Saxena S, Thornicroft G, Knapp M, Whiteford H. Resources for mental health: Scarcity, inequity, and inefficiency. Lancet. 2007;370(9590):878–889. 10.1016/S0140-6736(07)61239-2 17804062

[19] Jacob KS, Sharan P, Mirza I, et al. Mental health systems in countries: Where are we now? Lancet. 2007;370(9592):1061–1077. 10.1016/S0140-6736(07)61241-0 17804052

[20] Petersen I, Lund C, Bhana A, Flisher AJ. A task-shifting approach to primary mental health care for adults in South Africa: Human resource requirements and costs. J Ment Health Policy Econ. 2012;15(1):29–38. 10.1093/heapol/czr012 21325270

[21] Docrat S, Besada D, Cleary S, Daviaud E, Lund C. Mental health system costs, resources and constraints in South Africa: A national survey. Health Policy Plan. 2019;34(9):706–719. 10.1093/heapol/czz085 31544948 PMC6880339

[22] Van Rensburg TJ, De Jager S, Makrelov KH. Fiscal multipliers in South Africa after the global financial crisis. S Afr J Econ Manag Sci. 2022;25(1):a4191. 10.4102/sajems.v25i1.4191

[23] Health Systems Trust. South African Health Review 2022: Health systems recovery after COVID-19 [Internet]. Durban: Health Systems Trust; 2022 [cited 2025 Oct 9]. Available from: https://www.hst.org.za/publications/Pages/SAHR2022.aspx

[24] Gumede W. How lack of democracy fuels coups in Africa [Internet]. allAfrica. 2023 Sep 22 [cited 2025 Nov 11]. Available from: https://allafrica.com/stories/202309270378.html

[25] Lund C, Kleintjes S, Kakuma R, Flisher AJ. Public sector mental health systems in South Africa: Inter-provincial comparisons and policy implications. Soc Psychiatry Psychiatr Epidemiol. 2010;45(3):393–404. 10.1007/s00127-009-0078-5 19506789

[26] Wolvaardt GG, Stein DJ, Mumbauer AE. South Africa’s mental health human resource dilemma: From shortage to solution. S Afr Health Rev. 2024;27:142351. 10.61473/001c.142351

[27] Western Cape Department of Health. Healthcare 2030: The road to wellness [homepage on the Internet]. Cape Town: Western Cape DoH; 2014. Available from: https://d7.westerncape.gov.za/assets/departments/health/healthcare2030.pdf

[28] Western Cape Government: Health and Wellness. Provincial implementation plan for the national strategic plan in HIV, TB and STIs 2023–2028 [Internet]. Cape Town: Western Cape Government: Health and Wellness; 2023 [cited 2025 Sep 23]. Available from: https://groundup.org.za/media/uploads/documents/western_cape_hiv_plan.pdf

[29] Vala S. Registered counsellors’ experiences of their professional career development [master’s thesis on the Internet]. Bloemfontein: University of the Free State; 2017 [cited 2025 Nov 11]. Available from: https://scholar.ufs.ac.za/server/api/core/bitstreams/544cee1a-5017-49bf-9b87-196dbc8f7dd7/content

[30] Rouillard MCM, Wilson L, Weideman S. Registered counsellors’ perceptions of their role in the South African context of providing mental healthcare services. S Afr J Psychol. 2016;46(3):337–349. 10.1177/0081246315591340

[31] Joubert C, Hay J. Registered psychological training at a South African faculty of education: Are we impacting educational communities? S Afr J Educ. 2020;40(3):a1840. 10.15700/saje.v40n3a1840

[32] Palinkas LA, Horwitz SM, Green CA, Wisdom JP, Duan N, Hoagwood K. Purposeful sampling for qualitative data collection and analysis in mixed method implementation research. Adm Policy Ment Health. 2015;42(5):533–544. 10.1007/s10488-013-0528-y 24193818 PMC4012002

[33] Abel E, Louw J. Registered counsellors and professional work in South African psychology. S Afr J Psychol. 2009;39(1):99–108. 10.1177/008124630903900109

[34] Sorsdahl K, Petersen I, Myers B, Zingela Z, Lund C, Van der Westhuizen C. A reflection of the current status of the mental healthcare system in South Africa. Lancet Reg Health Africa. 2023;24:100624. 10.1016/j.lanafr.2023.100624

[35] Pillay AL. Psychology’s health and future in South Africa. S Afr J Psychol. 2016;46(2):149–154. 10.1177/0081246316642699

[36] Dube F, Uys LR. Integrating mental health care services in primary health care clinics: A survey of primary health care nurses’ knowledge, attitudes and beliefs. Prim Care S Afr Fam Pract. 2016;58(3):1–7. 10.1080/20786190.2016.1191747

[37] Mbedzi TE, Van der Wath AE, Moagi MM. Healthcare needs and expectations of family members caring for mental healthcare users in South Africa. Curationis. 2024;47(2):2625. 10.4102/curationis.v47i2.2625 39625089 PMC11621881

[38] Braun V, Clarke V. Toward good practice in thematic analysis: Avoiding common problems and becoming a knowing researcher. Int J Transgend Health. 2023;24(1):1–6. 10.1080/26895269.2022.2129597 36713144 PMC9879167

[39] Clarke V, Braun V. Teaching thematic analysis: Overcoming challenges and developing strategies for effective learning. Psychologist. 2013;26(2):120–123.

[40] Department of Health. Health Professions Act, 1974 (Act No. 56 of 1974): Regulations defining the scope of the profession of psychology (Notice No. R. 704). Pretoria: Government of South Africa; 2011 [cited 2025 Oct 9]. Available from: https://www.gov.za/sites/default/files/gcis_document/201409/34581rg9582gon704.pdf

[41] Smith JA, Flowers P, Larkin M. Interpretative phenomenological analysis: Theory, method and research. London: SAGE Publications; 2009.

[42] Republic of South Africa. Protection of Personal Information Act, No. 4 of 2013 (POPIA). Pretoria: Government Printer; 2013.

[43] South African College of Applied Psychology. SACAP – The role of registered counsellors in South Africa [Internet]. 2023 Sep 15 [cited 2025 Nov 11]. Available from: https://www.sacap.edu.za/gallery/sacap-the-role-of-registered-counsellors-in-south-africa/

[44] Creswell JW, Poth CN. Qualitative inquiry and research design: choosing among five approaches. 4th ed. Los Angeles (CA): SAGE Publications; 2018.

[45] Schleider JL. The fundamental need for lived experience perspectives in developing and evaluating psychotherapies. J Consult Clin Psychol. 2023;91(3):119–121. 10.1037/ccp0000798 36795434 PMC10033367

[46] Clement S, Schauman O, Graham T, et al. What is the impact of mental health-related stigma on help-seeking? A systematic review of quantitative and qualitative studies. Psychol Med. 2015;45(1):11–27. 10.1017/S0033291714000129 24569086

[47] Schnyder N, Panczak R, Groth N, Schultze-Lutter F. Association between mental health-related stigma and active help-seeking: Systematic review and meta-analysis. Br J Psychiatry. 2017;210(4):261–268. 10.1192/bjp.bp.116.189464 28153928

